# Inhaled 5‐HT_1B_ _/1D_ Receptor Antagonist Attenuates Sumatriptan‐Induced Sensitization of Capsaicin‐Sensitive Lung Vagal Afferents: Implications for Preventing Sumatriptan‐Associated Adverse Chest Symptoms

**DOI:** 10.1002/cph4.70121

**Published:** 2026-03-11

**Authors:** Nai‐Ju Chan, Zung Fan Yuan, Chang‐Chih Kuo, Chun‐Chun Hsu

**Affiliations:** ^1^ School of Respiratory Therapy, College of Medicine, Taipei Medical University Taipei Taiwan; ^2^ Graduate Institute of Medical Sciences, College of Medicine, Taipei Medical University Taipei Taiwan; ^3^ Department of Physiology School of Medicine, College of Medicine, Taipei Medical University Taipei Taiwan; ^4^ Department of Physiology and Master Program in Biomedical Sciences School of Medicine, Tzu Chi University Hualien Taiwan; ^5^ Division of Pulmonary Medicine, Department of Internal Medicine Taipei Medical University Hospital, Taipei Medical University Taipei Taiwan

**Keywords:** 5‐HT_1B/1D_ receptors, adverse chest effects, dyspnea, GR127935, lung sensory fibers, pulmonary drug administration, sumatriptan

## Abstract

**Background:**

Sumatriptan, an antimigraine drug, is known to cause adverse chest symptoms such as dyspnea and chest tightness. We previously showed that sumatriptan sensitized capsaicin‐sensitive lung vagal (CSLV) afferents in rats, a mechanism potentially underlying these symptoms. This sensitizing effect was abolished by intravenous GR127935, a 5‐HT_1B/1D_ receptor antagonist. Therefore, to develop a potential therapeutic strategy, we hypothesized that targeting GR127935 to the lungs, rather than delivering it systemically, could prevent sumatriptan‐induced chest discomfort by reducing CSLV‐afferent sensitization without compromising sumatriptan's antimigraine efficacy within the trigeminovascular system.

**Methods:**

Experiments were performed in anesthetized male Brown‐Norway rats. CSLV‐afferent excitability and afferent‐mediated airway reflexes were assessed using single‐fiber recordings and respiratory pattern monitoring. The antimigraine efficacy of sumatriptan was evaluated based on its inhibitory effect on dural plasma protein extravasation evoked by unilateral electrical stimulation of the trigeminal ganglion.

**Results:**

Intravenous sumatriptan potentiated capsaicin‐evoked CSLV‐afferent discharges and afferent‐mediated airway responses. However, pretreatment with inhaled GR127935 significantly blocked this potentiating effect. The sumatriptan's antimigraine efficacy was not affected by inhaled GR127935, but was reduced by intravenous GR127935. Consistently, intravenous, but not inhaled GR127935, altered sumatriptan‐induced hypotension, indicating that pulmonary delivery minimized systemic effects. Moreover, inhaled GR127935 did not change baseline cardiorespiratory parameters or fiber activity, suggesting it is unlikely to cause adverse cardiorespiratory effects.

**Conclusions:**

Inhalation of a 5‐HT_1B/1D_ receptor antagonist effectively prevented sumatriptan‐induced sensitization of CSLV afferents without diminishing sumatriptan's antimigraine efficacy. Our findings suggest that inhaled 5‐HT_1B/1D_ antagonists may offer a promising strategy to prevent or treat sumatriptan‐induced adverse chest symptoms.

## Graphical abstract

Our previous study suggests that sumatriptan‐induced chest discomfort is associated with hypersensitivity of CSLV afferents via 5‐HT_1B/1D_ receptors on nerve endings. The present study demonstrates that inhaled 5‐HT_1B/1D_ receptor antagonism suppresses this hypersensitivity without compromising antimigraine efficacy, supporting local pulmonary antagonism as an effective strategy to alleviate adverse chest effects. Created with Biorender.

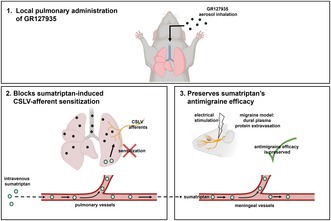

**Abbreviations**
5‐HT_1B/1D_ receptors: 5‐hydroxytryptamine 1B and 1D receptors
CNS: central nervous system
CSLV afferent: capsaicin‐sensitive lung vagal afferent
FA: fiber activity
HSD: honestly significant difference
LSD: least significance difference
REML: restricted maximum likelihood
T_E_
: expiratory time
TRPV1: transient receptor potential vanilloid 1

## 1 Introduction

Sumatriptan, a 5‐hydroxytryptamine 1B and 1D (5‐HT_1B/1D_) receptor agonist, is widely used as a first‐line therapy for acute migraine attacks (Belvís et al. 2009; Khan et al. 2021; Puledda et al. 2024). However, since its approval in 1991, its use has frequently been associated with chest discomfort, such as dyspnea and chest tightness (Hillis and Macintyre 1993; Visser et al. 1996; Ottervanger et al. 1997). Because sumatriptan has vasopressor effects (Razzaque et al. 1999) and these symptoms resemble angina (Dechant and Clissold 1992), early reports attributed them to myocardial ischemia and recommended caution in patients with cardiovascular disease (Dodick 2018). Nevertheless, sumatriptan‐induced adverse chest symptoms also occur in migraineurs without cardiovascular disease (Visser et al. 1996; Dahlöf and Mathew 1998; Lewis et al. 1997). Moreover, multiple assessments, including electrocardiography (Tomita et al. 2002; MacIntyre et al. 1993, 1992) and myocardial perfusion imaging (Lewis et al. 1997), have consistently shown normal findings even when chest symptoms were reported (Lewis et al. 1997; Tomita et al. 2002). These observations provide strong evidence against myocardial ischemia as the underlying cause. Thus, the mechanisms underlying sumatriptan‐induced chest symptoms remain unclear.

Capsaicin‐sensitive lung vagal (CSLV) afferents innervate the entire respiratory tract and lung periphery (Lee et al. 2003; Lee and Pisarri 2001; Lee and Yu 2014). These nociceptive‐like, highly chemosensitive fibers transmit sensory signals to the central nervous system (CNS) upon activation and evoke unpleasant respiratory sensations in humans (Chan et al. 2024; Lee 2009; Burki and Lee 2010a). In anesthetized rats, we recently showed that intravenous sumatriptan sensitized CSLV afferents, thereby amplifying stimulus‐evoked sensory traffic to the CNS (Chan et al. 2025). These findings support the hypothesis that, clinically, chest discomfort after oral or subcutaneous sumatriptan may occur when the absorbed drug enters the pulmonary circulation and sensitizes CSLV afferents. Furthermore, intravenous infusion of GR127935, a 5‐HT_1B/1D_ receptor antagonist, nearly abolished sumatriptan‐induced sensitization of CSLV afferents in rats (Chan et al. 2025), suggesting that 5‐HT_1B/1D_ receptors may represent a therapeutic target for mitigating the adverse chest symptoms associated with sumatriptan use. However, systemic administration (either oral or intravenous) of a 5‐HT_1B/1D_ receptor antagonist would likely blunt sumatriptan's antimigraine action within the trigeminovascular system, rendering this approach impractical.

Pulmonary drug delivery is an effective approach for treating airway symptoms and can reduce systemic side effects. We hypothesize that inhalation of a 5‐HT_1B/1D_ receptor antagonist would allow the drug to be primarily distributed within the lungs, thereby reducing its systemic concentration. This localized delivery may attenuate sumatriptan‐induced sensitization of CSLV afferents while preserving its antimigraine efficacy. In this study, we employed a model of neurogenic plasma protein extravasation in the dura mater, a key pathophysiological mechanism underlying migraine (Durham and Russo 2002; Wattiez et al. 2020), to evaluate the antimigraine efficacy of sumatriptan based on its anti‐neurogenic inflammatory effects. Accordingly, this study was conducted to determine whether inhaled GR127935 (1) suppresses sumatriptan‐induced sensitization of CSLV afferents in Brown‐Norway rats, (2) preserves sumatriptan's inhibitory effect on dural plasma protein extravasation, and (3) alters baseline cardiorespiratory parameters and CSLV‐afferent activity, particularly given the high local drug concentrations anticipated in the lungs.

## 2 Methods

### 2.1 Animals Preparation

Male Brown‐Norway rats were purchased from the National Center for Biomodels (Taipei, Taiwan). All experiments were conducted in accordance with the Guide for the Care and Use of Laboratory Animals issued by the National Institutes of Health and were approved by the IACUC (permit LAC‐2024‐0415) of Taipei Medical University. Rats were maintained in pairs per cage at the Laboratory Animal Center of Taipei Medical University, with a 12:12 h light/dark cycle (lights on at 07:00) and ad libitum access to food and water. All efforts were made to minimize the number of animals used and to reduce pain during the experiments. Anesthetics were administered prior to surgical procedures, and the depth of anesthesia was monitored throughout the experiments by pinching the rat's tail. Supplemental doses were provided as needed to sustain the elimination of pain reflexes. At the end of the experiments, animals were euthanized by intravenous injection of potassium chloride under deep anesthesia. A total of 75 Brown‐Norway rats were used in this study and were randomly assigned to control and experimental groups. All experimental procedures complied with the Animal Research: Reporting of In Vivo Experiments guidelines (Percie du Sert et al. 2020).

### 2.2 General Surgical Preparation

Adult Brown‐Norway rats weighing 250–350 g were used in this study. Anesthesia was induced via intraperitoneal injection of α‐chloralose (100 mg/kg, MilliporeSigma, St. Louis, MO, USA) and urethane (500 mg/kg, MilliporeSigma, St. Louis, MO, USA) dissolved in distilled water containing 2% borax (MilliporeSigma, St. Louis, MO, USA). After confirming a adequate depth of anesthesia for the surgical procedure, the animal was placed in a supine position on a thermostatically controlled surgical plate. Body temperature was maintained at 36°C throughout the procedures using a heating pad. A tracheal incision was made between the 2^nd^ and 3^rd^ C‐shaped cartilage rings below the thyroid cartilage, and an intratracheal tube was inserted for measuring breathing patterns and tracheal pressure. A catheter was inserted into the right jugular vein, with its tip advanced near the right atrium to allow rapid delivery of CSLV‐afferent stimulants into the pulmonary circulation. Another catheter was placed in the left femoral vein for administration of pharmacological agents or supplemental anesthetics. The right femoral artery was catheterized and connected to a pressure transducer for continuous monitoring of arterial blood pressure.

### 2.3 Electrophysiological Recording of CSLV Afferents

**FIGURE 1:**
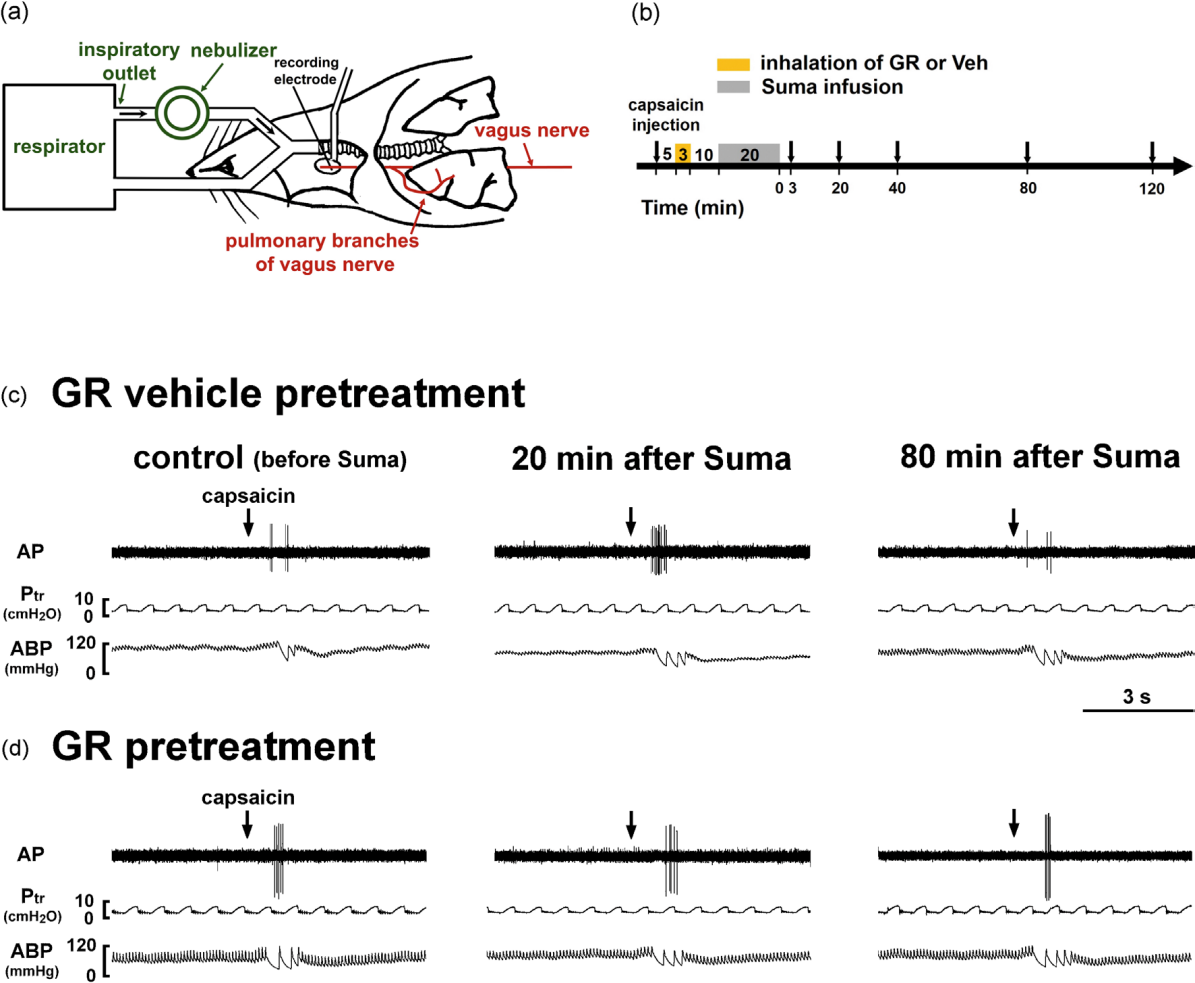
Inhalation of GR127935 (GR) aerosol inhibits sumatriptan (Suma)‐induced potentiation of the capsaicin‐sensitive lung vagal (CSLV)‐afferent responses to capsaicin injections in rats. (a) Schematic diagram illustrating the experimental setup for single‐fiber recording of CSLV afferents in an anesthetized, artificially ventilated rat. The afferent activity was recorded from a teased pulmonary filament, while GR127935 (GR) or vehicle (Veh) aerosol generated by an Aeroneb nebulizer was delivered into the lungs via a respirator during inspiration. (b) Experimental timeline illustrating CSLV‐afferent responses to right‐atrial capsaicin injections (arrows) obtained from 38 min before and 3, 20, 40, 80 and 120 min after termination of the infusion of Suma (1.2 mg/kg). The effects of pretreatment with GR or its vehicle aerosol, administered 10 min before Suma, were tested. (c and d) Representative recordings showing CSLV‐afferent responses to capsaicin injections (arrows) before (control), and at 20 min and 80 min after termination of the Suma infusion in two anesthetized rats pretreated with either GR (240 μg/mL for 3 min; lower panels) or Veh (isotonic saline; upper panels) aerosol. Note that pretreatment with GR aerosol abolished the sumatriptan‐induced potentiation of afferent responses to capsaicin. AP, action potential; Ptr, tracheal pressure; ABP, arterial blood pressure.

The measurement of fiber activity (FA) of CSLV afferents was described in detail in our previous study (Chan et al. 2025; Lin et al. 2020). A schematic diagram of the experimental setup for recording FA of CSLV afferents is presented in Figure 1a. Briefly, anesthetized rats were ventilated using a respirator (model 683; Harvard Apparatus, Holliston, MA, USA) with a tidal volume of 8 mL/kg and a respiratory frequency of 50 breaths/min to mimic the breathing pattern of anesthetized, spontaneously breathing rats (Table 2). Tracheal pressure was measured via a side port of the tracheal cannula using a differential pressure transducer (model P300D; Validyne, Northridge, CA, USA), and arterial blood pressure was measured via a femoral catheter connected to a pressure transducer (model P23XL; Spectramed, Columbus, OH, USA). A midline thoracotomy was performed to expose the thoracic cavity. Both vagus nerves were ligated just above the diaphragm to eliminate afferent inputs from visceral organs below the diaphragm. To maintain a near‐normal functional residual capacity, the expiratory outlet of the respirator was submerged under 3 cmH₂O pressure. The neck was incised along the midline, and approximately 1 cm of the right cervical vagus nerve was carefully separated from the adjacent common carotid artery. The nerve was sectioned as far rostrally as possible, and the distal (caudal) end was placed on a small dissecting platform immersed in mineral oil. From the desheathed nerve trunk, a fine filament was teased and positioned on a platinum‐iridium hook electrode for recording. Action potentials were amplified with an AC/DC differential amplifier (model 3000; A‐M Systems, Sequim, WA, USA), monitored via an audio monitor (model AM8RS; Grass Technologies, West Warwick, RI, USA), and displayed on an oscilloscope (model 2211; Tektronix, Beaverton, OR, USA). The filament was further teased until a single‐unit action potential was isolated. Signals were recorded and analyzed using a polygraph recorder (model MP36; Biopac, Goleta, CA, USA). CSLV afferents were identified by two criteria: (1) a robust response to capsaicin (2 μg/kg) within 2 s after a right‐atrial bolus injection via a jugular catheter, and (2) a response to gentle pressing of the lungs with a cotton swab at the end of the experiment to confirm fiber location. FA responses were calculated as the difference between peak and baseline FA values (ΔFA). Peak responses to capsaicin were averaged over 3 s after stimulation.

### 2.4 Measurement of Airway Reflexes

In anesthetized, spontaneously breathing rats, respiratory airflow was monitored using a heated pneumotachograph (model 8420; Hans Rudolph, Shawnee, KS, USA) coupled with a differential pressure transducer (model P300D; Validyne, Northridge, CA, USA). Tidal volume was calculated by integrating the measured airflow signal. Arterial blood pressure was continuously recorded via a femoral artery catheter connected to a pressure transducer (model P23XL; Spectramed, Columbus, OH, USA). All physiological signals were acquired using a polygraph recorder (model MP30; Biopac, Goleta, CA, USA) and analyzed with Biocybernetics TS‐100 software (Taipei, Taiwan). Prior to capsaicin injection, the lungs were hyperinflated to a tracheal pressure exceeding 20 cmH_2_O to standardize lung volume history. The apneic ratio was determined by dividing the longest expiratory time (T_E_) within 20 s after capsaicin injection by the baseline T_E_, calculated as the average of 10 breaths before injection.

### 2.5 Electrode Implantation Surgery Into the Trigeminal Ganglion

The paired nonconcentric bipolar electrode was made of two 55‐mm platinum‐iridium wires (model 778000; A‐M Systems, Sequim, WA, USA) insulated with Teflon (127.0 μm bare, 203.2 μm insulated). The wires were arranged in parallel and inserted into a stainless‐steel tube cut from a 22‐G syringe needle (inner diameter = 0.41 mm, length = 45 mm). The wire tips extended approximately 1 mm beyond the end of the stainless‐steel tube, whereas the opposite ends were soldered to plug pins for connection to an electrical stimulator (model 2100; A‐M Systems, Sequim, WA, USA). In anesthetized, spontaneously breathing rats, the head was placed in a stereotaxic frame (Stoelting Co., Wood Dale, IL, USA). A mid‐sagittal incision was made to expose the calvarium, and a 2‐mm‐diameter burr hole was drilled 3.2 mm lateral and 2.5 mm posterior to the bregma on the right side. The bipolar electrode was lowered 10.0 mm from the dura mater through the burr hole into the right trigeminal ganglion. Following electrode implantation, the assembly was fixed to the calvarium using dental acrylic and a screw.

### 2.6 Measurement of Plasma Protein Extravasation in the Dura Mater After Unilateral Electrical Stimulation of the Trigeminal Ganglion

Seven min before the right trigeminal ganglion was electrically stimulated (20‐min duration, 1.2‐mA current, 5‐ms pulse width, 5 Hz), Evans blue dye (80 mg/kg) was intravenously infused via femoral vein catheter. One hundred twenty min after the termination of electrical stimulation, isotonic saline was perfused through the left ventricle at a constant pressure of 120 mmHg for 10 min. Subsequently, the rats were decapitated, and bilateral dura mater tissues excluding the area covered on trigeminal ganglia were harvested and weighed (Figure 5a). Evans blue was extracted from the dura mater by incubation in 0.16 mL formamide (MilliporeSigma, St. Louis, MO, USA) at 80°C for 24 h. The optical density of the formamide extract was measured at 620 nm using a microplate reader (BioTek Epoch Microplate Spectrophotometer, Santa Clara, CA, USA) and quantified based on a standard curve of Evans blue ranging from 1 to 18 μg/mL. The severity of dural plasma protein extravasation was calculated as the ratio of Evans blue dye content (μg Evans blue/mg tissue) in the right dura mater (stimulated side) to that in the left dura mater (contralateral side).

### 2.7 Local Pulmonary Administration of a 5‐HT_1B_ _/1D_ Receptor Antagonist

GR127935 was selected in our study as a potential therapeutic agent to prevent sumatriptan‐induced sensitization of CSLV afferents. This choice was based on our previous findings showing that GR127935 antagonizes the 5‐HT_1B/1D_ receptors expressed on bronchopulmonary sensory neurons and inhibits sumatriptan‐induced sensitization (Chan et al. 2025). GR127935 aerosol was generated using an Aeroneb Nebulizer (Kent Scientific Corporation, Torrington, CT, USA) at a rate of 0.40–0.45 mL/min, with particle size ranging from 2.5 to 4.0 μm. In CSLV‐afferent electrophysiological recording experiments, the aerosol was delivered via the tracheal cannula into the lungs by a respirator. The nebulizer was connected to the breathing circuit between the inspiratory outlet of the respirator and the tracheal cannula (Figure 1a). In airway reflex and dural extravasation experiments, aerosols were actively inhaled via a tracheal tube by anesthetized, spontaneously breathing rats (Figure 3a). In all experiments, GR127935 aerosol inhalation was performed for 3 min and completed 10 min before the intravenous infusion of sumatriptan (1.2 mg/kg, 0.1 mL/min for 20 min).

### 2.8 Chemical Agents

Stock solutions of chemical agents were prepared as follows: sumatriptan succinate (10 mg/mL; United States Pharmacopeia, North Bethesda, MD, USA) was prepared in isotonic saline; capsaicin (250 μg/mL; MilliporeSigma, St. Louis, MO, USA) was prepared in 1% ethanol, 1% Tween 80, and 98% isotonic saline; phenylbiguanide (1 mg/mL; MilliporeSigma, St. Louis, MO, USA) was prepared in isotonic saline; GR127935 (1 mg/mL; Tocris, Bristol, UK) was prepared in distilled water; and Evans blue (40 mg/mL; MilliporeSigma, St. Louis, MO, USA) was prepared in isotonic saline containing 200 IU heparin (MilliporeSigma, St. Louis, MO, USA). Working solutions of these pharmacological agents at the desired concentrations were prepared daily by dilution with isotonic saline based on the animal's body weight.

### 2.9 Statistical Analysis

The minimal sample sizes for each group were determined to achieve a power of 0.8 and alpha = 0.05 calculated using G*Power software based on preliminary data. To ensure objectivity, data analyses and calculations were carried out in a blinded manner by two individual investigators. Statistical analyses were performed using Prism version 10 (GraphPad Software, San Diego, CA, USA). Before statistical analysis, Shapiro–Wilk tests were conducted to evaluate whether the data followed a normal distribution. When the data satisfied the normality assumption, statistical analyses for single‐factor comparisons were performed using a one‐way analysis of variance (ANOVA) followed by Tukey's Honestly Significant Difference (HSD) post hoc test or a two‐tailed paired *t*‐test. For two‐factor comparisons, two‐way ANOVA followed by Tukey's HSD post hoc test was applied. For datasets that did not meet the assumption of normality, single‐factor analyses were performed using the two‐tailed Wilcoxon signed‐rank test. For two‐factor analyses, a mixed‐effects model (restricted maximum likelihood, REML) followed by Tukey's HSD or Fisher's Least Significant Difference (LSD) post hoc test was used. A value of *p* < 0.05 was considered significant. All data are reported as the mean ± SEM. Statistical analyses, numbers of animals used (*n*), and *p* values are reported in figure legends.

## 3 Results

### 3.1 Inhalation of GR127935 Aerosol Inhibits Sumatriptan‐Induced Potentiation of CSLV‐Afferent Excitability to Capsaicin in Anesthetized, Ventilated Rats

**FIGURE 2:**
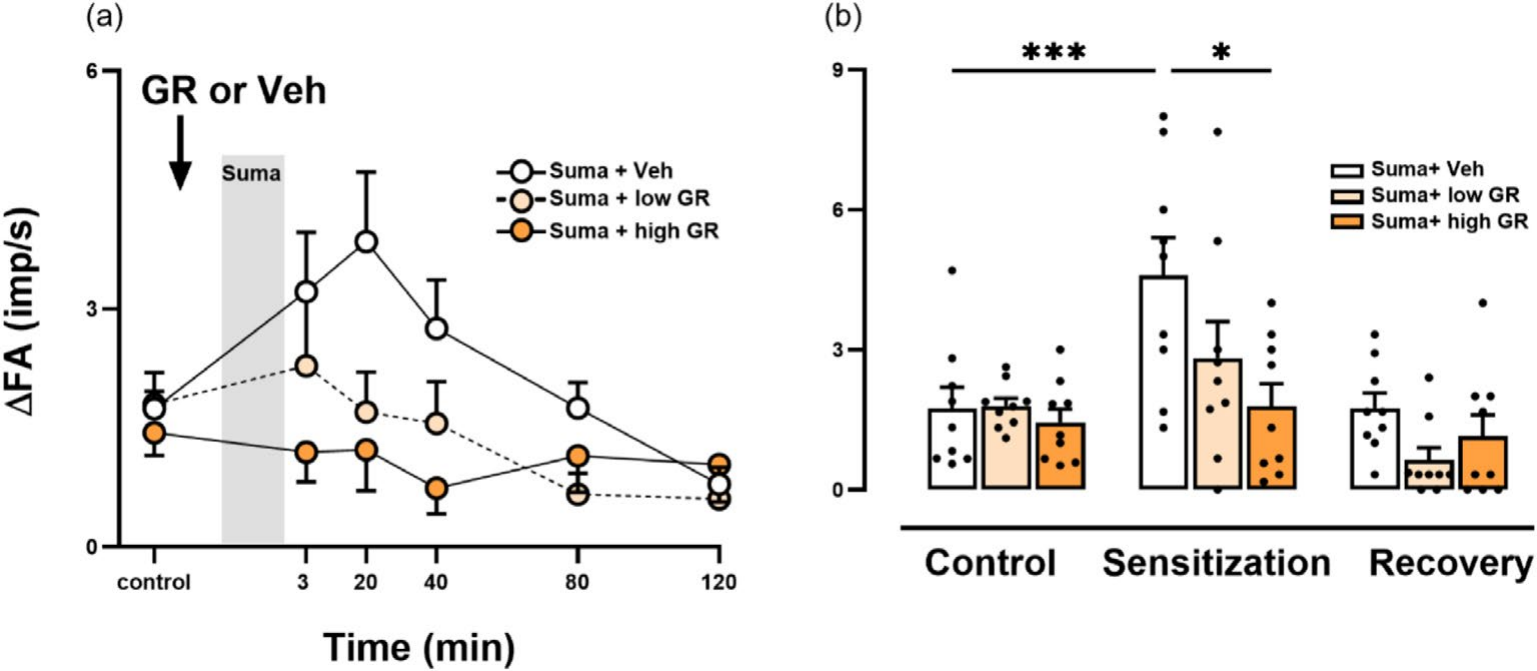
Inhalation of GR127935 (GR) aerosol concentration‐dependently blocks sumatriptan (Suma)‐induced potentiation of capsaicin‐elicited discharges of capsaicin‐sensitive lung vagal (CSLV) afferents. (a) CSLV‐afferent responses to capsaicin injections were measured before (control) and after a 20‐min Suma infusion (gray vertical bar) in anesthetized, artificially ventilated rats pretreated with vehicle (Veh; isotonic saline; white circles), low‐concentration (180 μg/mL; light‐orange circles) and high‐concentration GR (240 μg/mL; dark‐orange circles). (b) Group data of the increases in fiber activities (ΔFAs) in response to capsaicin collected at three time points: Before Suma infusion (control), at peak response time of 3 or 20 min (sensitization), and at 80 min after termination of Suma infusion (recovery). The white, light‐orange, and dark‐orange bars represent pretreatment with Veh, low‐concentration GR, and high‐concentration GR, respectively. ΔFA is the difference between the peak FA (averaged over a 3‐s interval) and the baseline FA for each fiber. Data are mean ± SEM (*n* = 9). *p < 0.05 and ***p < 0.005 using a mixed‐effects model followed by Tukey's HSD post hoc test.

To determine whether GR127935 aerosol inhalation inhibits sumatriptan‐induced sensitization of CSLV afferents, fiber responses to right‐atrial bolus injection of capsaicin [0.5 μg/kg; a transient receptor potential vanilloid 1 (TRPV1) agonist] were compared before and after intravenous infusion of sumatriptan (1.2 mg/kg). The effects of pretreatment with GR127935 aerosol or its vehicle via inhalation prior to sumatriptan infusion were tested (Figure 1b). Under control conditions (before sumatriptan infusion), capsaicin elicited mild discharges of CSLV afferents (Figure 1c; left panel). However, 20 min after termination of sumatriptan infusion (1.2 mg/kg), capsaicin‐elicited fiber discharges were markedly potentiated (Figure 1c; middle panel), and this potentiating effect recovered by 80 min later (Figure 1c; right panel). In sharp contrast, when GR127935 aerosol (240 μg/mL for 3 min) was delivered into the lungs via a respirator as a pretreatment, the sumatriptan‐induced potentiation was abolished (Figure 1d). As summarized in Figure 2, in the group pretreated with GR127935 vehicle, sumatriptan‐induced potentiation of capsaicin‐evoked discharges of CSLV afferents typically occurred at either 3 or 20 min after its infusion termination (Figure 2a). The peak response to capsaicin after sumatriptan infusion was approximately 2.63‐fold higher than the pre‐infusion level (*p* < 0.005; Figure 2b). In contrast, inhalation of GR127935 aerosol inhibited the sumatriptan‐induced potentiation in a concentration‐dependent manner (Figure 2b). For example, pretreatment with low‐concentration GR127935 reduced the sumatriptan‐induced potentiation from 2.63‐ to 1.57‐fold, whereas pretreatment with a high‐concentration GR127935 almost completely abolished this effect (1.24‐fold; *p* < 0.05) (Figure 2b). These findings suggest that GR127935 aerosol delivered into the lungs via a respirator prevents sumatriptan‐induced sensitization of CSLV afferents.

### 3.2 Spontaneous Inhalation of GR127935 Aerosol Inhibits Sumatriptan‐Induced Potentiation of CSLV Afferent‐Mediated Airway Reflexes in Anesthetized, Spontaneously Breathing Rats

**FIGURE 3:**
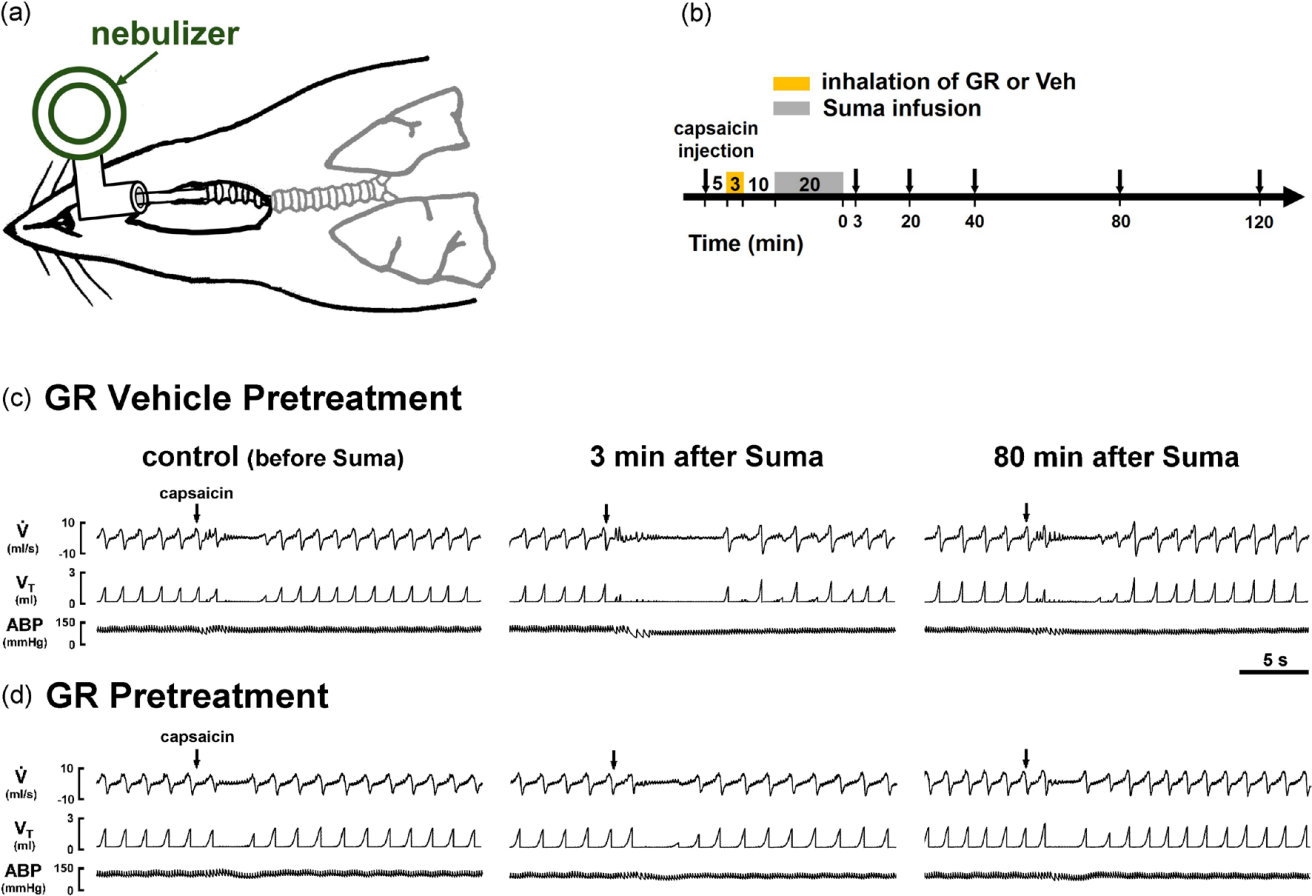
Spontaneous inhalation of GR127935 (GR) aerosol inhibits sumatriptan (Suma)‐induced potentiation of the apneic responses to capsaicin injections in rats. (a) Schematic diagram illustrating the experimental setup for spontaneous aerosol inhalation in an anesthetized rat. A tracheal cannula was connected to an Aeroneb nebulizer. (b) Experimental timeline illustrating apneic responses to right‐atrial capsaicin injections (arrows) obtained before and at several time points after termination of Suma infusion (1.2 mg/kg). The effects of pretreatment with GR or its vehicle (Veh) aerosol were tested. (c and d) Representative recordings showing apneic responses to capsaicin injections (arrows) before (control), and at 3 min and 80 min after termination of the Suma infusion in two anesthetized rats pretreated with either GR (240 μg/mL for 3 min; lower panels) or Veh (isotonic saline; upper panels) aerosol. Note that pretreatment with GR aerosol abolished the sumatriptan‐induced potentiation of apneic responses to capsaicin. $\dot{V,}$ airflow rate; V_T_, tidal volume; ABP, arterial blood pressure.

**FIGURE 4:**
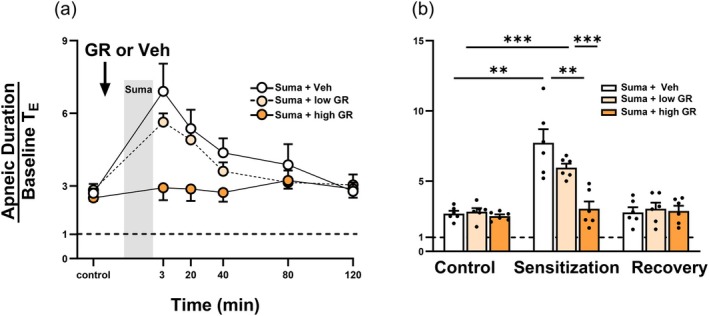
Inhalation of GR127935 (GR) aerosol concentration‐dependently blocks sumatriptan (Suma)‐induced potentiation of apneic responses to capsaicin injections. (a) Apneic responses to capsaicin injections were measured before (control) and after a 20‐min Suma infusion (gray vertical bar) in anesthetized, spontaneously breathing rats pretreated with vehicle (Veh; isotonic saline; white circles), low‐concentration (180 μg/mL; light‐orange circles) and high‐concentration GR (240 μg/mL; dark‐orange circles). (b) Group data showing the apneic responses to capsaicin collected at three time points: Before Suma infusion (control), at peak response times of 3 or 20 min (sensitization), and at 80 min after termination of Suma infusion (recovery). The white, light‐orange, and dark‐orange bars represent pretreatment with Veh, low‐concentration GR, and high‐concentration GR, respectively. Data are mean ± SEM (*n* = 6). The horizontal dashed line depicts an apneic ratio of 1 (indicating no apnea). **p < 0.01 and ***p < 0.005 using a two‐way ANOVA followed by Tukey's HSD post hoc test.

To mimic the clinical use of inhaled medications, GR127935 aerosol was delivered via spontaneous inhalation in anesthetized rats (Figure 3a). We then evaluated whether this pretreatment approach could inhibit the sumatriptan‐induced potentiation of airway reflexes mediated by central integration of CSLV‐afferent inputs (Figure 3b). Under control conditions (before sumatriptan infusion), a right‐atrial bolus injection of capsaicin (1.0 μg/kg) elicited a mild, transient apneic response, a hallmark reflex mediated by activation of CSLV afferents (Figure 3c; left panel). In a rat pretreated with the GR127935 vehicle aerosol, sumatriptan markedly potentiated apneic response to capsaicin injection at 3 min after termination of the infusion (Figure 3c; middle panel); this potentiating effect reversed by 80 min later (Figure 3c; right panel). In contrast, spontaneous inhalation of GR127935 aerosol (240 μg/mL for 3 min) before sumatriptan infusion abolished the potentiating effect induced by sumatriptan (Figure 3d). As summarized in Figure 4, apneic responses were measured before and after sumatriptan infusion in rats pretreated with GR127935 or vehicle aerosol. In groups pretreated with vehicle or low‐concentration GR127935 (180 μg/mL) aerosol, sumatriptan‐induced potentiation occurred at 3 or 20 min after infusion termination and returned to control levels by 80 min later; only pretreatment with high‐concentration GR127935 aerosol abolished this potentiating effect (vehicle vs. GR127935, *p* < 0.01; Figure 4b).

To exclude the possibility that the inhibitory effect of inhaled GR127935 on sumatriptan‐enhanced CSLV afferent‐mediated airway reflexes resulted from suppression of baseline stimulus‐evoked responses, additional control experiments were performed in animals not treated with sumatriptan. The reflexes mediated by CSLV afferents were evoked by capsaicin (a TRPV1 agonist) and phenylbiguanide (a 5‐HT_3_ receptor agonist). Inhalation of GR127935 aerosol did not alter these reflexes elicited by either stimulus (Figure S1). These findings indicate that inhaled GR127935 attenuates the sumatriptan‐induced potentiation of CSLV afferent‐mediated reflexes, rather than inhibiting baseline reflexes.

### 3.3 Spontaneous Inhalation of GR127935 Aerosol Does Not Affect the Inhibitory Effect of Sumatriptan on Dural Plasma Protein Extravasation in Anesthetized, Spontaneously Breathing Rats

**FIGURE 5:**
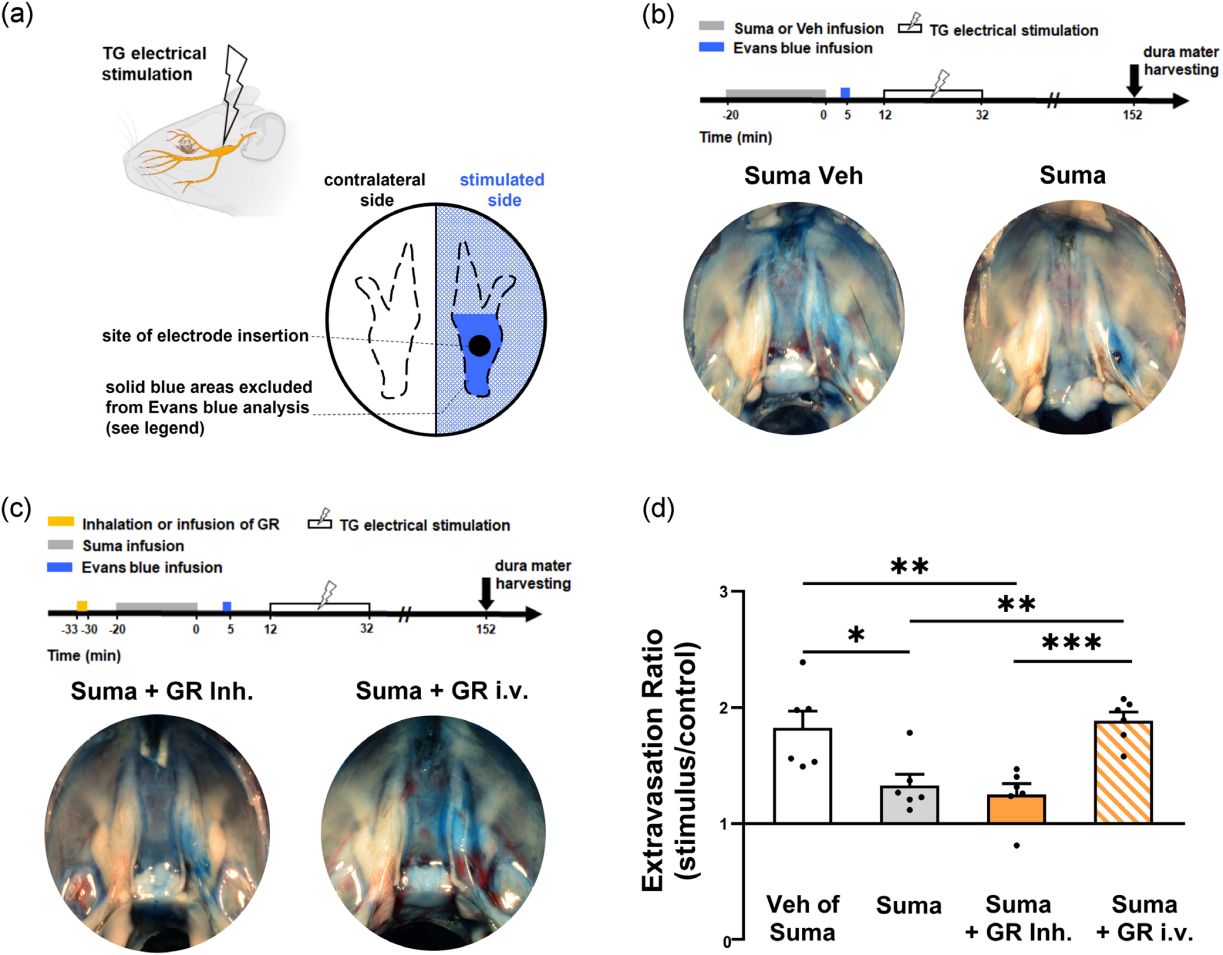
Inhaled, but not infused, GR127935 (GR) does not alter sumatriptan (Suma)‐induced inhibition of dural plasma protein extravasation in rats. (a) Upper panel: Schematic illustration of a migraine model induced by unilateral electrical stimulation of the trigeminal ganglion (TG) in an anesthetized, spontaneously breathing rat. Lower panel: Schematic diagram showing the Meckel's cave of rat after electrical stimulation. Plasma protein extravasation in the dura mater was assessed by measuring Evans blue content within the tissue. Solid blue areas were excluded from quantification to avoid possible interference from mechanical injury caused by electrode insertion. (b) Upper panel: Experimental timeline showing two groups receiving Suma (1.2 mg/kg) or its vehicle (Veh) 12 min before trigeminal electrical stimulation (20 min, 1.2 mA, 5 ms, and 5 Hz). Dura mater was harvested 120 min after termination of electrical stimulation. Lower panel: Meckel's cave of rat after pretreatment with Suma (right) or Veh (left) before electrical stimulation. (c) Upper panel: Experimental timeline showing two groups receiving GR via inhalation (Inh.; 240 μg/mL for 3 min) or intravenous (i.v.) infusion (300 μg/kg) 10 min before Suma infusion. Other experimental time points were identical to those described in Figure 5b. Lower panel: Meckel's cave of rat after pretreatment with GR via i.v. infusion or Inh. before Suma infusion and trigeminal ganglion stimulation. (d) Group data showing the inhibitory effect of Suma on trigeminal ganglion stimulation‐induced dural plasma protein extravasation, and the effects of pretreatment with GR via Inh. or i.v. routes on this inhibition. Data are mean ± SEM (*n* = 6). *p < 0.05, **p < 0.01, ***p < 0.005 using a one‐way ANOVA followed by Tukey's HSD post hoc test.

In previous experiments, aerosolized GR127935 administered via inhalation effectively alleviated the respiratory adverse effects induced by intravenous sumatriptan. However, it remains uncertain whether a portion of the inhaled GR127935 is absorbed through the pulmonary vasculature and subsequently enters the systemic circulation, thereby potentially influencing the therapeutic efficacy of sumatriptan within the trigeminovascular system. Therefore, we next evaluated the effect of GR127935 aerosol inhalation on sumatriptan‐induced suppression of neurogenic plasma protein extravasation in the dura mater (Figure 5a). In an anesthetized, spontaneously breathing rat, plasma protein extravasation in the dura mater induced by unilateral electrical stimulation of the trigeminal ganglion (1.2 mA, 5 ms, 5 Hz, 20 min) was markedly attenuated by pretreatment with intravenous infusion of sumatriptan (1.2 mg/kg), whereas vehicle pretreatment had no such effect (Figure 5b), indicating an anti‐neurogenic inflammatory action of sumatriptan. GR127935 (300 μg/kg) administered via systemic intravenous infusion reversed the inhibitory effect of sumatriptan on dural plasma protein extravasation (Figure 5c; lower right panel). In sharp contrast, spontaneous inhalation of GR127935 aerosol (240 μg/mL) did not alter the inhibitory effect of sumatriptan (Figure 5c; lower left panel). As summarized in Figure 5d, sumatriptan significantly reduced the plasma protein extravasation in the dura mater induced by electrical stimulation of the trigeminal ganglion by approximately 60% (*p* < 0.05). This inhibitory effect of sumatriptan was not affected by pretreatment with GR127935 administered via the inhalation route, but was reversed when GR127935 was administered via the infusion route (*p* < 0.01). These findings suggest that pulmonary delivery of GR127935 minimizes its distribution to the trigeminovascular system, thereby preserving the inhibitory effect of sumatriptan on neurogenic plasma protein extravasation in the dura mater.

### 3.4 Spontaneous Inhalation of GR127935 Aerosol Does Not Affect Cardiovascular Responses to Sumatriptan in Anesthetized, Spontaneously Breathing Rats

**FIGURE 6:**
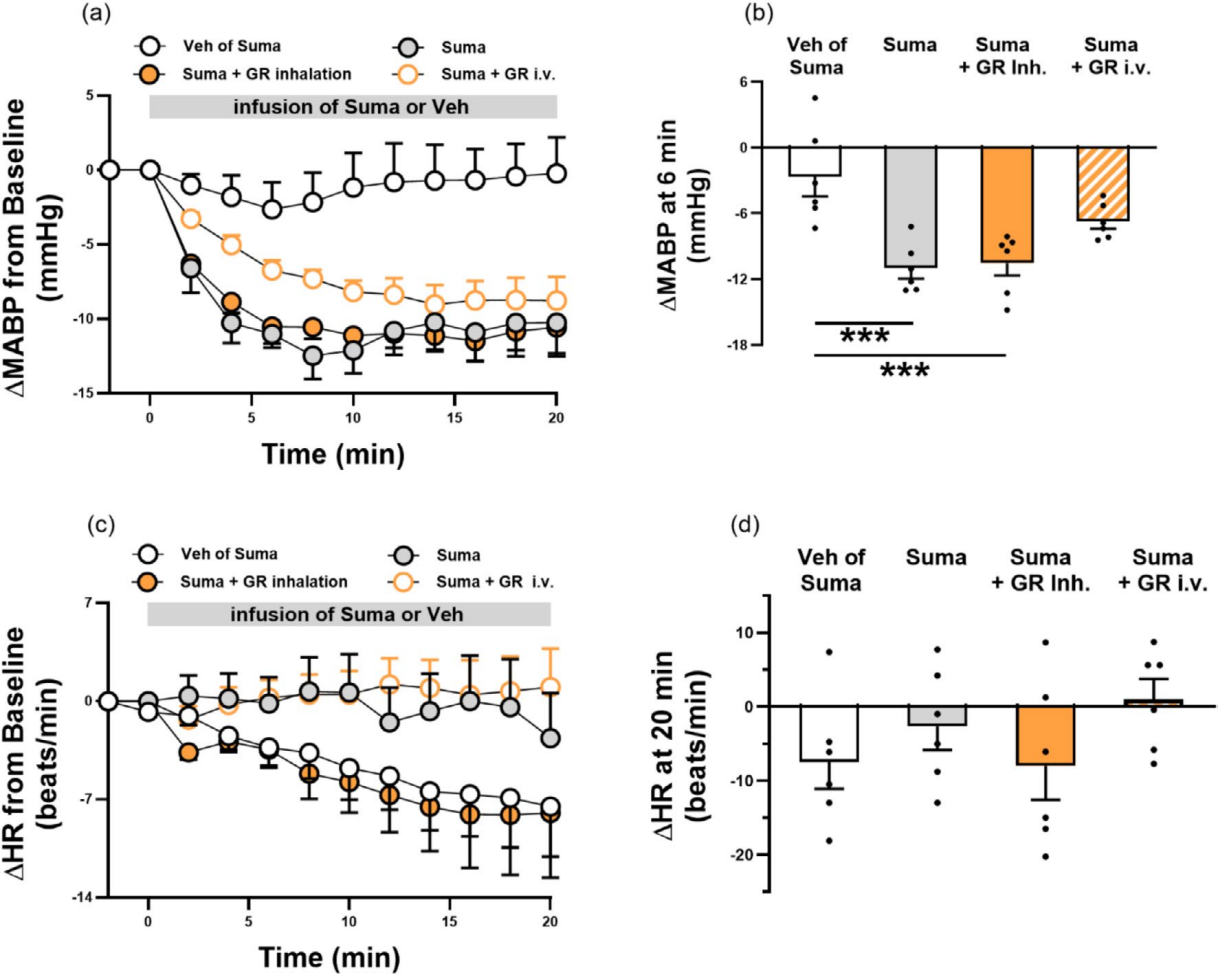
Pretreatment with GR127935 (GR) by inhalation (Inh.), but not by intravenous (i.v.) infusion, alters the pattern of sumatriptan (Suma)‐induced hypotension in rats. (a) Histogram showing changes in mean arterial blood pressure (ΔMABP, expressed as the difference from baseline) during a 20‐min infusion of Suma (gray filled circles) or its Veh (white open circles) in anesthetized, spontaneously breathing rats. The effects of pretreatment with GR administered via Inh. (orange filled circles) or i.v. infusion (orange open circles) on Suma‐induced blood pressure changes were also examined. (b) Group data showing ΔMABP at 6 min after infusion onset of Suma or Veh, with pretreatment by GR via Inh. or i.v. infusion before Suma infusion. (c and d) Histogram (left panel) and group data (right panel) showing the effects of Suma or its Veh on changes in heart rate (ΔHR, expressed as the difference from baseline). The effects of pretreatment with GR, administered via Inh. or i.v. infusion, on Suma‐induced HR changes were also examined. Peak ΔHR responses were obtained from 20 min after Suma or its Veh infusion. Data are mean ± SEM (*n* = 6). ***p < 0.005 using a one‐way ANOVA followed by Tukey's HSD post hoc test.

Consistent with previous findings (Pagniez et al. 1998), our results showed that in anesthetized, spontaneously breathing rats, intravenous infusion of sumatriptan (1.2 mg/kg) caused an immediate decrease in arterial blood pressure. The maximal reduction, approximately 12 mmHg, occurred 6 min after the onset of infusion and persisted until the infusion was terminated. In contrast, sumatriptan vehicle infusion did not elicit any hypotensive effect (Figure 6a). We next determined whether administration of GR127935 could alter the hypotension induced by sumatriptan. The results showed that intravenous infusion of GR127935 (300 μg/kg) did not affect the peak magnitude of the sumatriptan‐induced decrease in blood pressure but delayed the time to reach this peak from 6 min to 14 min after infusion onset (Figure 6a). In contrast, such a delay was not observed when GR127935 was administered via the inhalation route (240 μg/mL for 3 min) (Figure 6a). As summarized in Figure 6b, sumatriptan infusion significantly decreased mean arterial blood pressure compared with its vehicle at 6 min after infusion onset (*p* < 0.005). This hypotensive effect was also observed in rats pretreated with inhlaed, but not intravenous, GR127935 (*p* < 0.005). On the other hand, heart rate changes did not differ significantly among the four groups (Figure 6c,d). These findings suggest that administration of GR127935 via inhalation minimizes its diffusive systemic effects.

### 3.5 Airway Exposure to Aerosolized GR127935 Does Not Alter Baseline Cardiorespiratory Parameters or CSLV‐Afferent Activity in Anesthetized Rats

We further determined whether inhalation of GR127935 aerosol could alter physiological parameters, particularly in the lungs, where its concentration is expected to be highest. Compared with baseline cardiorespiratory parameters and fiber activities obtained from 1 min before GR127935 aerosol inhalation (240 μg/mL for 3 min), no significant changes were observed at 5 min after the termination of inhalation in anesthetized rats (Tables 1 and 2).

**TABLE 1:** Effect of inhalation of GR127935 (GR) or its vehicle on baseline tracheal pressure (Ptr) and fiber activity (FA) in anesthetized, artificially ventilated rats.

	Vehicle inhalation		GR inhalation	
	Before	After	Before	After
Ptr (cmH_2_O)	8.19 ± 0.21	8.40 ± 0.22	8.04 ± 0.17	8.35 ± 0.31
FA (imp/s)	0.04 ± 0.03	0.02 ± 0.02	0.04 ± 0.03	0.07 ± 0.03

*Note:* Baseline values were averaged over a 10‐s period immediately before and 5 min after termination of GR inhalation (240 μg/mL for 3 min) or its vehicle (isotonic saline). No significant differences were found in either baseline Ptr or FA before and after inhalation. Data are mean ± SEM (*n* = 9). Statistical analysis was performed using a mixed‐effects model followed by Fisher's LSD post hoc test.

Abbreviation: imp, impulses.

**TABLE 2:** Effect of inhalation of GR127935 (GR) or its vehicle on baseline tidal volume (V_T_), respiratory frequency (*f*_R_), mean arterial blood pressure (MABP), and heart rate (HR) in anesthetized, spontaneously breathing rats.

	Vehicle inhalation		GR inhalation	
	Before	After	Before	After
V_T_ (mL)	1.94 ± 0.07	1.96 ± 0.09	1.81 ± 0.08	1.77 ± 0.07
*f*_R_ (breaths/min)	47 ± 2	46 ± 1	48 ± 2	48 ± 2
MABP (mmHg)	104 ± 3	104 ± 2	105 ± 3	99 ± 4
HR (beats/min)	357 ± 4	352 ± 4	366 ± 7	365 ± 8

*Note:* Baseline values were averaged over a 10‐s period immediately before and 5 min after termination of GR inhalation (240 μg/mL for 3 min) or its vehicle (isotonic saline). No significant differences were found in these cardiorespiratory parameters before and after inhalation. Data are mean ± SEM (*n* = 6). Statistical analysis was performed using a mixed‐effects model followed by Fisher's LSD post hoc test.

## 4 Discussion

### 4.1 Major Findings and Clinical Relevance

This study demonstrates that local pulmonary delivery of GR127935 prevented sumatriptan‐induced sensitization of CSLV afferents while preserving the antimigraine efficacy of sumatriptan in rats. Our results showed that in anesthetized rats, respirator‐assisted administration of aerosolized GR127935 completely abolished the sumatriptan‐induced sensitization of CSLV afferents in a concentration‐dependent manner (Figures 1 and 2). Similarly, spontaneous inhalation of GR127935 aerosol, mimicking the clinical administration of inhaled medications, suppressed the sumatriptan‐induced enhancement of airway reflexes mediated by activation of CSLV afferents projecting to the CNS (Figures 3 and 4). These findings suggest that inhaled GR127935 effectively mitigates sensitization of CSLV afferents and airway hypersensitivity caused by sumatriptan. Importantly, in our established rat migraine model, inhalation of GR127935 aerosol did not interfere with the antimigraine efficacy of sumatriptan (Figure 5). In addition, GR127935 inhalation did not cause significant changes in cardiorespiratory parameters, including respiratory patterns, blood pressure, heart rate, and tracheal pressure (Tables 1 and 2), suggesting a favorable safety and tolerability profile. Together with our recent findings showing that sumatriptan induces sensitization of CSLV afferents, which may contribute to the development of sumatriptan‐induced chest discomfort (Chan et al. 2025), the present study further demonstrates that localized pulmonary administration of a 5‐HT_1B/1D_ receptor antagonist may represent a potential therapeutic strategy for alleviating these chest side effects.

### 4.2 Mechanisms of Chest Discomfort and the Potential Role of CSLV Afferents

Chest discomfort, including dyspnea and chest tightness, is a subjective and unpleasant sensation generated by the projection of afferent signals to the CNS (Chan et al. 2024; Lee 2009; Burki and Lee 2010a; Fukushi et al. 2021). In general, the development of chest discomfort is attributed to local pathophysiological changes within tissues, which are subsequently detected by sensory nerves. For example, dyspnea associated with heart failure (Colucci et al. 2000; Yancy et al. 2013) or bronchoconstriction (Mahler and O'Donnell 2015). Another common mechanism, however, does not involve local pathophysiological changes but instead results from the direct action of chemicals on sensory nerve endings. A well‐recognized example is intravenous adenosine, a drug used clinically to treat arrhythmias, which is commonly associated with dyspnea as a side effect (Burki et al. 2005). Clinical studies have shown that this adenosine‐induced dyspnea, which occurs without bronchospasm (Burki et al. 2006), can be markedly attenuated by inhaled lidocaine through anesthesia of pulmonary sensory nerves (Burki et al. 2008; Burki and Lee 2010b), suggesting a critical role of these afferents. Furthermore, animal experiments have provided evidence that adenosine activates CSLV afferents (Hong et al. 1998; Kwong et al. 1998) and bronchopulmonary neurons (Chuaychoo et al. 2006), supporting the conclusion that the dyspneic side effect of adenosine treatment is mediated by its direct action on CSLV afferents. It is likely that a comparable mechanism underlies the chest discomfort observed following sumatriptan administration. Our recent study demonstrated that sumatriptan sensitized CSLV afferents and isolated neurons via 5‐HT_1B/1D_ receptors (Chan et al. 2025). These findings suggest that targeting CSLV‐afferent sensitization may represent a promising therapeutic strategy for alleviating chest discomfort.

### 4.3 Advantages of Inhaled GR127935 as a Treatment for Sumatriptan‐Induced Chest Discomfort

There is currently no convincing evidence that sumatriptan‐induced chest discomfort arises from local pathophysiological changes within the tissue. Consequently, no effective pharmacological treatments are currently available for these unpleasant respiratory sensations. Previous studies have suggested that chest discomfort of various origins can be attenuated by reducing central and/or sensory sensitivity, such as with oral opioids (Mahler and O'Donnell 2015; Mahler 2013) or inhaled local anesthetics (e.g., lidocaine) (Burki et al. 2008; Burki and Lee 2010b; Lee et al. 1993; Taguchi et al. 1991). However, these anesthetics also suppress critical airway defensive functions mediated by CSLV afferents, such as the cough reflex (Svajdova et al. 2019), and opioids may further cause adverse central effects (Mahler and O'Donnell 2015; Mahler 2013). By contrast, our present findings demonstrate that inhaled GR127935 blocked sumatriptan‐induced sensitization of CSLV afferents through antagonism of 5‐HT_1B/1D_ receptors (Figures 1 and 2); importantly, it did not alter the apneic responses mediated by TRPV1 and 5‐HT_3_ receptors (Figure S1), suggesting that essential physiological functions of CSLV afferents are preserved. Collectively, this pharmacological specificity highlights inhaled GR127935 as a promising and selective therapeutic strategy for suppressing sumatriptan‐induced chest discomfort.

### 4.4 Safety Considerations for Inhaled GR127935 Antagonist

Because inhalation of GR127935 causes the highest local drug concentrations in the lungs, potential pulmonary adverse effects remain uncertain. Although exogenous 5‐HT_1B_ receptor agonists induce contraction of isolated pulmonary vessels in rats (Keegan et al. 2001) and humans (Dodick 2004), long‐term oral administration of GR127935 in rodents does not alter pulmonary artery morphology or vascular contractility (Keegan et al. 2001), suggesting that 5‐HT_1B/1D_ receptors do not play a significant role in regulating pulmonary vascular tone under basal conditions. In addition, our results showed that airway exposure to GR127935 did not affect respiratory frequency, tidal volume, or tracheal pressure (Tables 1 and 2), all of which are highly sensitive indicators of pulmonary pathophysiology, such as inflammation or bronchoconstriction. Taken together, these observations suggest that inhaled GR127935 is unlikely to cause pulmonary adverse effects in rats. However, clinical studies are necessary to further confirm its safety and tolerability.

### 4.5 Gradual Loss of GR127935‐Mediated Inhibition of Sumatriptan‐Induced Hypotension During Sustained Infusion

Our study showed that sumatriptan infusion induced significant hypotension. However, when administered via intravenous infusion, GR127935 inhibited this hypotensive response by approximately 40% during the early phase of sumatriptan infusion (6 min after onset); this inhibitory effect gradually declined as the sumatriptan infusion continued (Figure 6). The mechanism underlying the progressively diminishing inhibition of sumatriptan‐induced hypotension by GR127935 remains unclear. However, this is unlikely to be due to insufficient antagonist efficacy, as our recent study showed that GR127935 maintained inhibition of CSLV‐afferent sensitization for at least 20 min after termination of sumatriptan infusion (Chan et al. 2025). Instead, the progressively diminishing effect of GR127935 may result from the involvement of non‐5‐HT_1B/1D_ receptor mechanisms as the cumulative plasma concentration of sumatriptan increases during its infusion. This hypothesis is further supported by studies in anesthetized rats showing that low‐dose sumatriptan‐induced hypotension can be attenuated by either a 5‐HT_1B/1D_ receptor antagonist (GR127935) or a 5‐HT_1A_ receptor antagonist (WAY100635). At higher doses, however, the hypotensive response is predominantly mediated by 5‐HT_1A_ receptor activation (Pagniez et al. 1998). Collectively, these findings suggest that sumatriptan‐induced hypotension involves both 5‐HT_1B/1D_ receptor‐dependent and ‐independent pathways.

Although our experiments did not identify the specific sites of 5‐HT_1B/1D_ receptor activation responsible for sumatriptan‐induced hypotension, previous studies have suggested that sumatriptan may act on prejunctional 5‐HT_1B/1D_ receptors located on sympathetic efferent terminals, thereby reducing sympathetic outflow (Morán et al. 1998; Watts et al. 2012; González‐Hernández et al. 2023). Furthermore, the observation that inhaled GR127935 did not alter the pattern of sumatriptan‐induced hypotension compared with systemic infusion suggests that inhaled GR127935 has minimal diffusive systemic effects.

### 4.6 Limitations of This Study

This study has two primary limitations. First, only male rats were used as experimental subjects, despite evidence that both the prevalence of migraine (Vetvik and MacGregor 2017) and the incidence of sumatriptan‐induced chest discomfort (Visser et al. 1996; Ottervanger et al. 1997) are significantly higher in women. Female rats were excluded because fluctuations in plasma estrogen levels across the estrous cycle can introduce substantial variability in sensitivity of CSLV afferents (Huang et al. 2018). Therefore, evaluating these effects in female rats would require ovariectomized models with estrogen supplementation to control for hormonal variability. This approach would substantially increase the animal numbers required; this was therefore beyond the scope of the present study. Nevertheless, the potential influence of sex‐related differences requires further investigation in future work. Second, aerosolized GR127935 was delivered to rat lungs via a tracheal cannula, which may have overestimated the therapeutic efficacy of inhaled agents by bypassing the upper‐airway barriers that are present during clinical inhalation. Although an oral inhalation animal model would better replicate clinical conditions, it does not allow us to measure afferent activity and respiratory parameters. In the present study, administration via a tracheal cannula represented the most appropriate delivery approach. It remains uncertain whether the complete inhibitory effect of GR127935 inhalation observed in this study can be fully translated to clinical conditions. Future studies aiming for a more comprehensive assessment of therapeutic efficacy should establish oral inhalation models that incorporate real‐time respiratory monitoring and fiber‐activity measurements.

## 5 Conclusions

Inhaled administration of a 5‐HT_1B/1D_ receptor antagonist minimized its systemic effects, thereby not only inhibiting sumatriptan‐induced sensitization of CSLV afferents but also preserving the antimigraine efficacy of sumatriptan within the trigeminovascular system. Moreover, inhalation of the receptor antagonist did not alter baseline cardiorespiratory parameters and afferent activity, indicating favorable safety and tolerability. Given that sumatriptan‐induced sensitization of CSLV afferents may contribute to the chest discomfort associated with sumatriptan use, we propose that pretreatment with inhaled 5‐HT_1B/1D_ receptor antagonist may represent a novel and practical strategy to prevent these adverse chest symptoms.

## Author Contributions

N.‐J.C. and C.‐C.H. designed research studies; N.‐J.C. and C.‐C.H. conducted experiments; N.‐J.C. acquired data and analyzed data; Z.F.Y., C.‐C.K., and C.‐C.H. interpreted results of experiments; N.‐J.C. and C.‐C.H. wrote the manuscript. All authors read and approved the final manuscript.

## Funding

This work was supported by the National Science and Technology Council, R.O.C, NSTC 114‐2320‐B‐038‐056; Taipei Medical University, TMU 112‐5431‐002‐400.

## Ethics Statement

Animals were handled in accordance with standards established by the Guide for the Care and Use of Laboratory Animals published by the National Institutes of Health, and approved by the IACUC (permit LAC‐2024‐0415) at Taipei Medical University. All experimental procedures complied with the ARRIVE guidelines.

## Conflicts of Interest

The authors declare no conflicts of interest.

## Supporting information


**Figure S1:** cph470121‐sup‐0001‐FigureS1.docx.

## Data Availability

The data that support the findings of this study are available from the corresponding author upon reasonable request.
