# Supplementary material for: Inhaled 5‐HT1B /1D Receptor Antagonist Attenuates Sumatriptan‐Induced Sensitization of Capsaicin‐Sensitive Lung Vagal Afferents: Implications for Preventing Sumatriptan‐Associated Adverse Chest Symptoms
Source: Compr Physiol. 2026 Mar 11;16(2):e70121. doi: 10.1002/cph4.70121 (PMC12980051; doi:10.1002/cph4.70121)
Supplement: Supplementary file 1 — Figure S1: cph470121‐sup‐0001‐FigureS1.docx. [file CPH4-16-e70121-s001.docx]

**
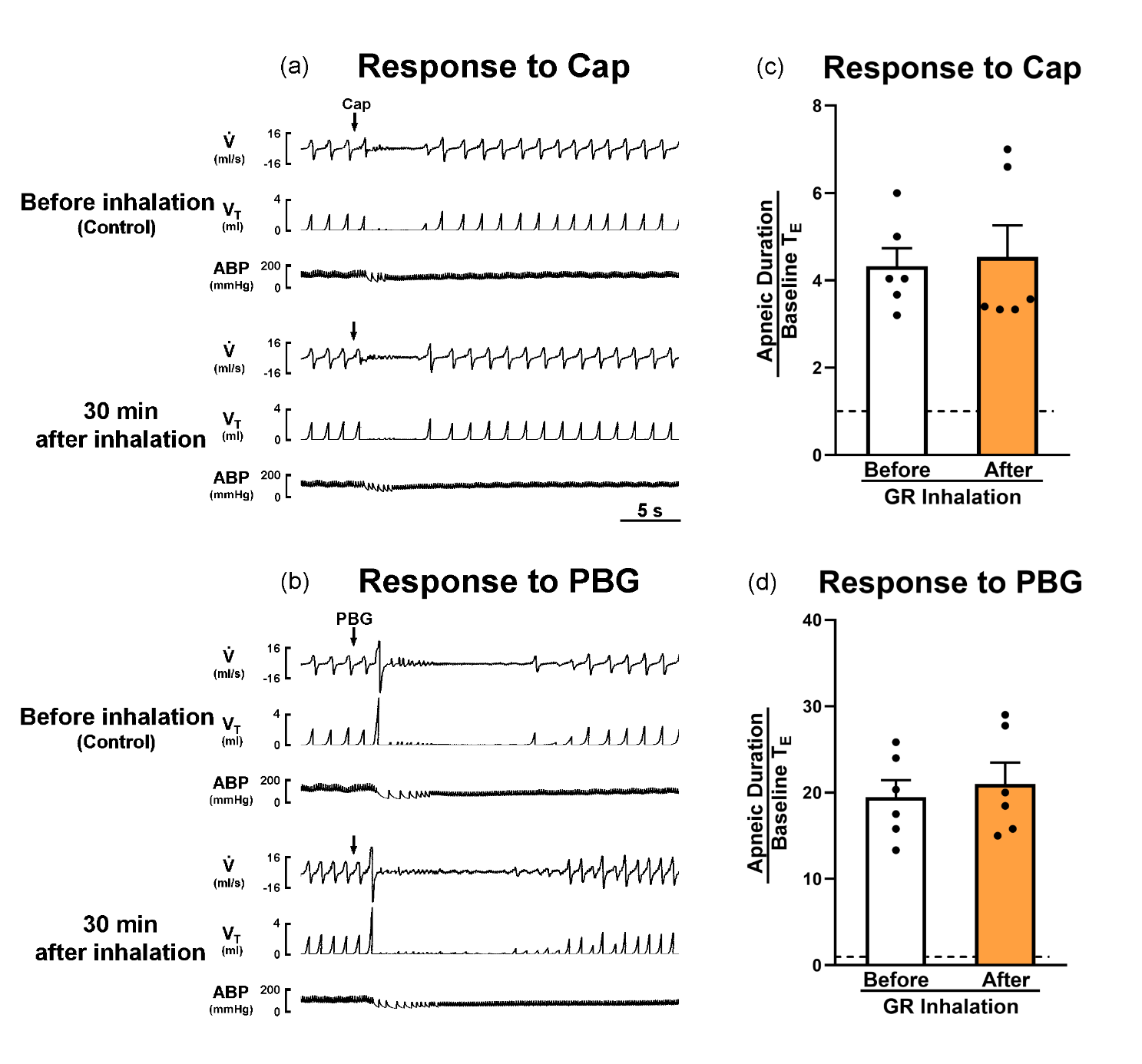
**

**Supplementary Figure 1. GR127935 (GR) aerosol does not alter apneic responses to capsaicin (Cap) and** **phenylbiguanide (PBG) injections in rats.
(a and b)** Representative recordings showing apneic responses to Cap (a; arrows) and PBG (b; arrows) injections measured before (control) and 30 min after termination of GR inhalation in an anesthetized, spontaneously breathing rat. Note that inhalation of GR aerosol did not alter the apneic responses triggered by capsaicin or PBG. **(c and d)** Group data showing apneic responses to Cap (c) and PBG (d) measured before (white bars) and 30 min after (orange bars) GR inhalation. Data are mean ± SEM (n = 6). The horizontal dashed line depicts an apneic ratio of 1 (indicating no apnea). Statistical analyses were performed using a two-tailed paired t-test for (c) and a two-tailed Wilcoxon signed-rank test for (d). $\dot{V,}$ airflow rate; V_T_, tidal volume; ABP, arterial blood pressure.
